# Gene drive systems: do they have a place in agricultural weed management?

**DOI:** 10.1002/ps.5137

**Published:** 2018-09-17

**Authors:** Paul Neve

**Affiliations:** ^1^ Biointeractions & Crop Protection Department Rothamsted Research, West Common Hertfordshire UK

**Keywords:** gene drive, weed management, direct genetic control, herbicide resistance, CRISPR–Cas9

## Abstract

There is a pressing need for novel control techniques in agricultural weed management. Direct genetic control of agricultural pests encompasses a range of techniques to introduce and spread novel, fitness‐reducing genetic modifications through pest populations. Recently, the development of CRISPR–Cas9 gene editing has brought these approaches into sharper focus. Proof of concept for CRISPR–Cas9‐based gene drives has been demonstrated for the control of disease‐vectoring insects. This article considers whether and how gene drives may be applied in agricultural weed management, focusing on CRISPR–Cas9‐based systems. Population‐suppression drives might be employed to introduce and proliferate deleterious mutations that directly impact fitness and weediness, whereas population‐sensitizing drives would seek to edit weed genomes so that populations are rendered more sensitive to subsequent management interventions. Technical challenges relating to plant transformation and gene editing *in planta* are considered, and the implementation of gene drives for timely and sustainable weed management is reviewed in the light of weed population biology. The technical, biological, practical and regulatory challenges remain significant. Modelling‐based studies can inform how and if gene drives could be employed in weed populations. These studies are an essential first step towards determining the utility of gene drives for weed management. © 2018 The Author. *Pest Management Science* published by John Wiley & Sons Ltd on behalf of Society of Chemical Industry.

## 1 INTRODUCTION

The potential for human‐mediated, genetic interventions to aid in the suppression and control of insect populations that negatively impact agriculture and human health has been recognized since the 1940s (for reviews see Leftwich *et al*. and Gould^1^, ^2^). However, during the last 5 years, these discussions have been given increased fervour with the development of the CRISPR–Cas9 system for genome editing,^3^ and the recognized potential to adapt this technology to drive precisely edited genes through wild populations.^4^ Although the potential application of gene drive systems for the reversal of resistance to herbicides has been highlighted,^4^, ^5^, ^6^, ^7^, ^8^ to date, there is no systematic review of the potential applications and constraints of these systems for weed management. This article briefly considers the historical development of genetic control technologies for insect pests, including recent proofs of concept for synthetic gene drives that control insects vectoring human disease. I consider the need for novel genetic control strategies in agricultural weed management and, in that context, the potential application, practicability, limitations and constraints of gene drives. I conclude with a call for modelling‐based studies to: (i) determine if these approaches have a practical application; and (ii) inform the future design and implementation of gene drive systems for weed management.

## 2 A ROLE FOR GENETIC CONTROL IN PEST MANAGEMENT

In agriculture and health care, the control of pest species (herbivorous and disease‐vectoring insects, pathogens and weeds) has become dominated by the application of synthetic pesticides. This approach has met with notable success, significantly reducing crop yield losses and saving lives through the enhanced control of insect vectors of human disease. However, intense selection has resulted in the widespread evolution of pesticide resistance,^9^, ^10^, ^11^ and there are increasing concerns about the off‐target environmental and human health impacts of pesticide use, meaning that novel, alternative control strategies are urgently needed. A variety of physical, agronomic, biological and agro‐ecological methods are available for pest control and population suppression, but the possibility of designing genetic control strategies that directly manipulate the genomes of pest species to reduce their fitness remains an intriguing and increasingly attainable goal.

### 2.1 Indirect genetic control of agricultural pests

In agriculture, it is possible to achieve indirect genetic control (Table A1) of pests through cultivar selection, breeding or genetic engineering (or editing) of crop germplasm. Many crop species and their wild relatives harbour genetic variation for host plant resistance to insect pests^12^ and pathogens.^13^ Crop varieties may also vary in their competitive^14^ and allelopathic potential^15^ against weeds. Where traits conferring enhanced tolerance, or even resistance to pests and pathogens have been bred out of modern crop varieties, these traits can be re‐introduced through marker‐assisted breeding, assuming that they have no deleterious agronomic and nutritional impacts.

One of the most transformative agricultural technologies of recent decades has been the development and commercialization of transgenic crops that express novel herbicide resistance and pest resistance traits.^16^ Transgenic herbicide‐resistant crops have revolutionized weed management by enabling the use of broad‐spectrum, non‐selective herbicides for weed control in crops.^17^ Crops have also been genetically engineered to produce insecticidal proteins for insect pest control, eliminating or reducing the need for insecticide applications. Although still prone to the evolution of pest resistance, and, in the case of transgenic herbicide‐resistant crops, still dependent on the use of herbicides, these technologies demonstrate the pest control advances that can be made with increased access to insights, tools and resources from molecular biology.

Several potential methods for the direct genetic control of insect pests have been proposed and developed, and these are reviewed in Section 2.2. However, although there have been some successes, in general, these have been limited by the significant difficulty of trying to proliferate engineered genes that reduce individual and population level fitness, through widely dispersed natural populations.

### 2.2 A brief history of direct genetic control for insect pests

In general terms, two types of approach have been suggested for direct genetic control of insects.^1^ Self‐limiting mechanisms rely on inundative introductions of modified insects that mate with local populations leading to population suppression through lethal matings. Self‐sustaining approaches rely on mechanisms that drive genetic alterations through populations. Typically, these ‘driven’ genetic alterations reduce fitness and/or pest status and increase in frequency via multigenerational, non‐Mendelian inheritance, potentially leading to population replacement. A number of mechanisms have been proposed that can limit the temporal and spatial spread of self‐sustaining drives.

#### 2.2.1 *Self‐limiting mechanisms*

Self‐limiting genetic mechanisms for insect control rely on the mass rearing of insects that are subsequently released into natural populations leading to sterile matings. Because these methods rely on lethality, they do not persist and spread in populations, and the degree of population suppression achieved depends on the proportion of matings between introduced and wild insect populations. The first insect genetic control system to be developed and deployed was the ‘sterile insect technique’.^18^ This approach relies on the mass‐rearing of insects, and their irradiation to induce sterility. A mass release of sterile insects (preferably males) is made into an area and matings between sterile males and wild females result in inviable embryos, reducing population numbers. This technique has been used for the control of seven species,^19^ but aside from some notable successes,^20^ the sterile insect technique has not risen to prominence because its success relies on targeting a relatively small pest population, on the capacity to mass‐rear insects, and may be hampered by the fitness burden carried by irradiated males.^2^

More recently, the ‘release of insects carrying a dominant lethal’ (RIDL) technique has been developed.^21^ This approach overcomes the need for irradiation by using transgenesis to introduce a lethal mutation into the genome of mass‐reared insects. The female‐specific RIDL system results in female mortality through sex‐specific alternative splicing that leads to the production of a tetracycline‐repressible transactivation fusion protein.^22^ A unique feature of this system is that lethality can be repressed if tetracycline is present in the diet, enabling the mass‐rearing of insects that carry and spread conditionally lethal mutations in the wild.

A third approach for the self‐limiting population suppression of insects has been termed the ‘incompatible insect technique’.^1^, ^23^ This approach, whose precise mechanisms are unknown, relies on the introduction of the maternally transmitted endosymbiotic bacteria*, Wolbachia* into laboratory‐infected mosquitoes. When *Wolbachia*‐infected males mate with uninfected females, the resulting embryos are inviable, providing the potential for the mass release of infected males as an alternative strategy that relies on neither irradiation nor transgenic approaches. Where *Wolbachia* is used for self‐limiting population control, it is important that only infected males are released because matings with infected females are compatible, providing a means to drive *Wolbachia* infections through populations (see Section 2.2.2). The incompatible insect technique has been trialled in a range of cage experiments and small‐scale field trials to affect population control for disease‐vectoring *Culex* and *Aedes* species.^23^

#### 2.2.2 *Self‐sustaining mechanisms*

The fundamental challenge for the design of self‐sustaining, direct genetic manipulation of pest genomes rests with the need to spread fitness‐reducing genes through widely dispersed, wild populations. With normal Mendelian inheritance, the fitness costs associated with those novel alleles would dictate against their spread through populations leading to, at best, their maintenance at low frequencies for limited periods and ultimately to their being purged from populations. However, the discovery in nature of several genetic mechanisms that distort or subvert the normal ‘rules’ of Mendelian inheritance offers some tantalizing possibilities for driving genetic modifications through pest genomes.

These naturally evolved mechanisms of ‘gene’ or ‘meiotic’ drive have been reviewed extensively elsewhere.^1^, ^23^, ^24^ One of the first recognized gene drive systems relied on the use of *Wolbachia* infection to drive phenotypes such as cytoplasmic incompatibility through populations. In the presence of *Wolbachia*‐infected males, infected females have a substantial fitness advantage, because uninfected females produce sterile offspring following matings with infected males. This leads to an overall reduction in mating success, while increasing the frequency of *Wolbachia* in fertile offspring, effectively driving *Wolbachia* through populations.^1^

The Y drive system can also distort sex ratios in insect populations via breakage of the X chromosome during meiosis. In *Aedes aegypti*, a segregation distorter linked to the Y chromosome is inherited by 80–90% of progeny in natural populations.^25^ Mobile transposable elements offer another means to introduce and proliferate novel genetic sequences throughout pest genomes. If insects could be genetically engineered so that a fitness‐reducing gene could be embedded with a transposon, that gene could be spread through pest populations.^26^ Another selfish genetic element, *Medea* has evolved naturally in beetles, fungi and plants.^27^ The *Medea* system causes the death of all embryos from a mating between a carrier and a susceptible individual, except those individuals that inherit the *Medea* element.

The existence in nature of a diversity of gene drive systems offers promise for their practical manipulation, and models have shown that these approaches have the potential to succeed in suppressing pest populations and limiting the transmission of insect‐vectored disease. However, field implementation is lacking, perhaps because of the difficulty of spreading genes that severely impact the fitness of pest populations. To date, the sterile insect technique, RIDL and *Wolbachia*‐based approaches remain the only genetic control strategies that have been utilized for practical pest management.

A considerable surge in interest in the application of gene drive systems has been catalysed by the suggestion that it may be possible to use homing endonuclease genes for the genetic control of wild populations of insect pests.^28^ In theory, if a homing endonuclease gene could be inserted into a functional gene, using transgenic approaches, it would result in loss of function of that gene. Initially, the loss of function would be heterozygous. However, in cells that are heterozygous for the presence of these homing genes, an enzyme cleaves the homologous chromosome in an identical position and inserts a copy of itself, converting a heterozygote into a homozygote. These selfish genes occur in a variety of microbes, usually situated in introns and therefore normally having no impact on gene function. The inherent homing, cleaving and copying machinery of these gene drives makes it theoretically possible to drive edited or engineered genes through the genomes of wild species, even when the resulting genetic changes reduce the fitness of recipient individuals and populations.

## 3 THE DEVELOPMENT OF CRISPR–Cas9 GENE DRIVE SYSTEMS

The discovery, and subsequent demonstration that the bacterially derived CRISPR–Cas9 system could be adapted for precise gene editing in eukaryotic cells^3^, ^29^ provided further promise for the development of gene drives in wild pest populations.^4^ The advantages of this system are that the Cas9 nuclease can be directed to cut almost any part of the genome, being directed to targeted sequences by a guide RNA. In applications of this technology for genome editing, the cut site is repaired using the homologous recombination pathway to copy the gene drive sequence at the cut site. The gene drive element consists of the guide RNA, Cas9 nuclease and an edited repair template that may incorporate a newly functional gene at the cut site or introduce precise sequence changes to the cut gene.^4^ In self‐sustaining gene drive systems, the sequences for the Cas9, the guide RNA and any edited or novel gene sequences that are included as ‘cargo’ are inserted into the genome at the location where the cut is made. In a diploid organism, the CRISPR–Cas9 is then automatically guided to cut the same site on the homologous chromosome and inserts the construct by homologous recombination. The modified organism therefore becomes homozygous for CRISPR–Cas9 and the edited gene, meaning that all gametes will transmit that construct. Matings between homozygous‐edited and wild‐type individuals will result in heterozygotes and, as before, the Cas9 will be guided to cut the homologous chromosome and insert the edited gene, leading to a mutagenic chain reaction.^30^ More recently, as concerns have risen over the potential limitless spread of self‐sustaining gene drives, split‐drive systems have been proposed whereby the gene drive depends on the presence of second gene that is inherited normally, limiting the spatial and temporal spread of gene drives.^7^, ^31^

### 3.1 Molecular, ecological and evolutionary constraints on CRISPR–Cas9 gene drives

The second part of this article focuses on the potential application of CRISPR–Cas9‐based gene drive system to weed management. In doing so, it is necessary to consider the molecular, ecological and evolutionary factors that enable and constrain successful gene drives in weed species (see Gould^2^ for a detailed account of the various biological factors impacting the feasibility of genetic control of pest species). Here, these factors are presented in general terms as they relate to the feasibility, fidelity, rate of spread (temporal and spatial), and evolutionary stability of gene drives. In following sections, they are considered with specific reference to the biology of weedy plants.

The first limiting step is the availability of a genetic transformation system for the target organism that is compatible with CRISPR–Cas9 gene editing. Where this is not a limitation, the molecular genetic potential for population‐level gene drives will depend on several factors related to homing efficiency.^4^ The Cas9 nuclease must reliably cut the genome at the target sequence; successful attempts at Cas9 genome editing in a wide range of species suggest that this should not be a fundamental constraint.^32^, ^33^ The specificity of cutting is important where precise gene editing or knockout is required, or where some insertion sites may lead to severe fitness penalties. This is harder to achieve in large genomes due to the potentially larger number of similar, off‐target sequences. Most critically for successful copying and propagation of the gene drive, it is important to ensure that the cut sequence is repaired by homologous recombination and not via the non‐homologous end‐joining pathway. The relative frequency of homologous versus non‐homologous DNA repair pathways may vary between organisms and between tissues, and the rate of homologous repair will set inherent limits on the propagation of the gene drive element.^4^ Attempts to design endonuclease gene drives in various insect species have resulted in successful copying following > 97% of cuts in mosquitoes,^34^ whereas similar efforts in fruit flies resulted in < 78% success.^35^ Ensuring high rates of homologous DNA repair in weed species is a necessary first step towards demonstrating the potential application of gene drive systems.

Even given a high percentage of conversion of heterozygote to homozygote individuals, gene drives take multiple generations to spread through populations.^36^ The rate at which gene drives can spread depends on several demographic and life history parameters. For example, drives spread more quickly when: (i) large numbers of individuals are introduced relative to the size of the established population; (ii) fitness costs associated with the drive are low; (iii) the generation time is short; (iv) targeted species are obligate outcrossers; and (v) rates of gene flow, mediated by pollen dispersal and seed movement are relatively high (Section 5).

As with all control strategies that significantly reduce pest fitness, gene drives are subject to counteracting evolutionary forces that operate to restore fitness and sustain viable populations. Evolution of resistance to gene drives may operate through mechanisms that overcome the molecular machinery for recognition, cutting and copying at edited sites or via selection of alleles in other parts of the genome that enable the restoration of fitness through compensatory evolution.^37^

## 4 APPLICATION OF CRISPR–Cas9 GENE DRIVES TO WEED CONTROL

**Figure 1:**
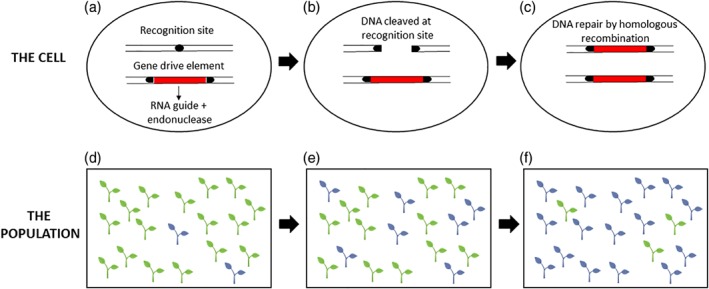
A CRISPR–Cas9 gene drive system for an agricultural weed. At the cellular level following a successful mating between introduced and wild‐type individuals, plants will carry the gene drive system in a heterozygous state (a). An RNA guide will direct the Cas9 nuclease to cut the DNA at the recognition site on the wildtype chromosome (b). The cut will be repaired by homologous recombination using the drive chromosome as a template and converting the individual to a homozygous state for the drive (c). At the population level, individuals with the engineered drive (blue plants) would be introduced into a wild population (green plants) (d) and would spread over time (e, f) until individuals carrying the drive allele dominate the population (adapted from Godfray *et al*. 50 and Frey and Malik 51.

The precept for CRISPR–Cas9‐based population suppression of weed species rests with the assumption that gene drives can be used to introduce and spread a fitness load that can limit the establishment, abundance, dispersal, persistence and/or impact of weed populations (Fig. 1).

This might be achieved via genetic manipulations that target weed traits relating to competitiveness, seed dormancy and persistence, phenology and morphology, although the potential of these approaches is currently limited by incomplete understanding of the molecular genetic basis of weed traits. Notwithstanding this, it is conceivable that homologues of the *Rht‐1* dwarfing genes that have been utilized in wheat breeding^38^ could be identified in related grass weeds. If these genes could be driven through populations, they may reduce weed competitive ability. Similarly, studies in weedy rice have begun to unravel the genetic basis of the evolved seed dormancy and seed‐shattering traits that contribute to the persistence and spread of wild rice, providing another molecular target for efforts to reduce the fitness of weeds in agroecosystems.^39^ Efforts could also be directed towards meiotic drive systems that bias the sex ratio in dioecious weedy species such as *Amaranthus* spp., leading to biased sex ratios. Other attempts to target plant reproduction and fecundity might focus on genetic manipulations that interfere with, for example, gametogenesis to limit pollen or ovule production, leading to reductions and/or biases in gamete production. The emerging field of weed genomics has recognized the value in research to determine the molecular genetic basis of ‘weediness’ traits to better inform how those traits evolve and as a potential novel source of genetic variation for crop improvement.^40^ Given the potential for direct genetic control of weed species, these efforts gain greater impetus if gene drive systems can be employed to knockout or modify weedy traits in wild populations.

Although there are likely to be geographical and biological limits to the spread of introduced population suppression‐based gene drives, these could, in theory, if they were to spread unchecked, lead to species extinction.^4^ Recognizing the potential ethical, regulatory and biological concerns associated with unmitigated population‐suppression drives, several authors have proposed strategies for safeguarding this technology to limit spatial and temporal spread.^4^, ^7^, ^31^ Of these, sensitizing drives, which focus on increasing the target population's susceptibility to subsequent management or environmental interventions, appear to offer greater potential for regulatory approval and subsequent application in weed control.

Sensitizing drives have the considerable advantage that population suppression is not contingent on the drive element itself, but on subsequent management, the application of which can be controlled in time and space. Sensitizing drives can be designed to spread genetically engineered genes that have no inherent negative consequences on organismal fitness, aiding their spread, but more importantly, ensuring that population suppression is conditional on specific management interventions. The most obvious application of sensitizing drives in weed management would be to revert herbicide‐resistant populations back to herbicide sensitivity. Because most herbicide targets are well known and many of the mutations that confer resistance have been characterized,^11^ this is technically feasible (notwithstanding the need to develop transformation systems for weed species and the technical challenges posed by adapting the CRISPR–Cas9 system for genome editing in those species). Driving susceptibility through weed populations to the point at which effective control of previously resistant populations would be possible would take several generations (see Section 5), during which the target herbicide could not be used because it would effectively kill plants carrying the elements propagating the reversion. Even low frequencies of resistant individuals being maintained in populations following a successful drive event could lead to the rapid evolution of resistance upon the recommencement of herbicide use. Subsequent management of newly sensitive populations would have to pay heed to carefully designed resistance management strategies. It should also be acknowledged that the application of this approach is complicated by the increasing recognition that many widespread mechanisms of herbicide resistance are conferred by complex, possibly polygenic mechanisms whose genetic determination and genetic architecture have not been fully resolved.^41^

Sensitizing drives could also be used to introduce changes into highly conserved, essential plant genes rendering them sensitive to specifically designed herbicidal molecules,^4^ by combining synthetic chemistry with synthetic and structural biology. Edited genes would have to maintain their normal enzymatic activity to effectively spread through populations, but these approaches could pave the way for the design of highly targeted chemical interventions that limit environmental and non‐target impacts.

Other intriguing possibilities for sensitizing drives can be imagined. Recently, weed control technologies that target and destroy mature weed seeds, limiting seed bank replenishment, have been developed.^42^ These technologies rely on the retention of weed seeds on mature plants at the time of crop harvest to enable their collection, removal and/or destruction. For some species, such as the major annual grass weed of north‐west Europe, *Alopecurus myosuroides*, early seed shattering limits the potential for these approaches. A gene drive that targeted seed‐shattering loci, leading to the retention of seeds on parent plants until crop harvest, could be combined with harvest weed control technology to offer a new method of control for this and other intractable weed species. Conceptually this is possible, as single nucleotide polymorphisms in target genes have been responsible for the loss of the shattering phenotype during the domestication of rice.^43^

## 5 WEED GENE DRIVES: THEORY INTO REALITY?

This article attempts to address the question ‘do gene drive systems have a place in agricultural weed management?’ In the preceding section, the aim was to demonstrate that a number of potential applications exist, particularly for sensitizing gene drives. The technical barriers are large, but conceptually at least, they are not insurmountable. These barriers include the development of transformation systems for weed species that are compatible with successful genome editing using CRISPR–Cas9 machinery and the identification of suitable molecular targets. As discussed in Section 3.1, the ultimate success of gene drives for weeds depends on overcoming molecular genetic constraints and challenges, but also ecological and evolutionary (population biology) barriers. The successful design and implementation of gene drives depends on collaboration between molecular biologists, weed ecologists, modellers and evolutionary biologists.

What would be the essential characteristics of a successful gene drive for weeds? Edited genes would need to spread through populations to high enough frequencies to affect sufficient population control within a reasonable time frame. The system would have to be deployed in a way to maximize evolutionary robustness. Modelling studies to explore the time frames over which gene drives could spread edited genes to fixation in natural populations have indicated 10–20 generations, depending on the fitness consequences (selection coefficient) of the edited gene and the conversion efficiency of a CRISPR–Cas9 homing and editing reactions.^37^ This model makes a number of assumptions that would likely be violated in weed populations infesting agroecosystems and a number of authors have noted the need for modelling studies to explore the potential of gene drives for agricultural pest management.^4^, ^6^ There are a number of specific characteristics of plant (weed) species that would likely moderate the potential for effective gene drives, and these are discussed in greater detail below.

### 5.1 The population biology of weed gene drive systems

Many major annual weed species produce a single generation per year, which imposes a significant constraint on the time it will take for gene drives to spread through populations. Unckless *et al*.^36^ predicted 10–20 generations to fix driven genes in wild populations when the initial frequency of edited individuals released into populations was 0.001. Weed populations are large; a moderate infestation of a major weed species would have an average seed bank density of 100 seeds m^−2^, meaning that a 10 ha cropping field would contain 10 million individuals (10^7^), requiring the release of 10^4^ seeds to achieve the spread rates simulated by Unckless *et al*.^36^ (all else being equal) in a single infested field. A reduction in the number of generations required to fix edited genes could be achieved with a higher release rate, but this would be challenging practically. In particular, the production of sufficient seeds containing the gene drive for releases at this scale would pose a significant technological and practical constraint because, compared with most insect pests, weedy plants have a long generation time, increasing the time required to multiply gene edited populations for release.

The Unckless *et al*.^36^ model also assumes that gene drives spread through a panmictic population. This simplifying assumption will be violated in many weed populations. Although several major weed species are outcrossing, pollen dispersal distances are limited, potentially slowing the spread of gene drives through populations. Within agricultural fields, this constraint could be somewhat mitigated by ‘releasing’ edited individuals throughout the field, probably through intentional mixing with the sown crop seed. The potential for the spread of gene drives would be severely limited in selfing weed species or in species with a perennial life cycle and gene drives would not be possible in species that can reproduce asexually, for example through vegetative propagation. Even for obligate outcrossing species, there will be a strong tendency towards the evolution of selfing in response to drives based on homing endonuclease genes.^44^ Many plant species have mixed mating systems and gene drives would likely bias populations towards increased rates of inbreeding. Finally, it would be important to consider the possibility for assortative mating between the local and introduced (gene edited) populations, particularly where, for example, there were differences in the timing of flowering, anthesis and pollen dispersal.

Another defining characteristic of many agricultural weed species, the possession of a persistent, dormant seed bank, also needs to be considered. Effectively, this means that only a small to moderate proportion of the viable weed population exists at any time as adult, reproductive plants. In this situation, the seed bank acts as a genetic ‘reservoir’ of individuals that are only exposed to the GDS following germination and growth to reproductive maturity. In species with long‐lived seed banks, this reservoir has the potential to significantly slow the spread of genetic manipulations through populations.

In summary, the potential success and application of gene drives for agricultural weeds will depend on a range of breeding system, genetic and life history parameters, and on the availability of genomic resources and plant transformation systems. Considering the application of gene drives for management of a range of major, global, herbicide‐resistant weeds species, it becomes evident that gene drives have application for some, but not all major species (Table 1).

**Table 1:** Summary of gene drive potential for major, global agricultural weeds

Species	Mating system	Vegetative	Fecundity	Seed bank persistence	Resistance risk	Ploidy	Genome	Distribution	Potential / priority
*Amaranthus palmeri*	Outcrossing (dioecious)	No	High	Low	Very high	Diploid	Medium, sequenced	North and South America	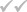
*Amaranthus tuberculatus*	Outcrossing (dioecious)	No	High	Medium	Very high	Diploid	Medium, congener sequenced	North America	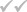
*Alopecurus myosuroides*	Outcrossing	No	Medium	Medium	Very high	Diploid	Large, no sequence	Europe,Asia	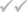
*Lolium rigidum*	Outcrossing	No	Medium	Medium	Very high	Diploid	Large, congener sequenced	Australia, Europe, South America, Africa	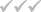
*Kochia scoparia*	Outcrossing	No	Medium	Low	Intermediate	Diploid	Medium, sequenced	North America	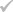
*Avena fatua*	Selfing	No	Low	Medium	Very high	Hexaploid	Large, no sequence	North and South America, Australia, Europe, Africa, Asia	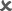
*Conyza canadensis*	Selfing	No	Medium	Medium	High	Diploid	Small, sequenced	North America, Europe, Asia	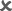
*Sorghum halepense*	Selfing	Yes	Medium	Low	Intermediate	Diploid and tetraploid	Small, congener sequenced	North and South America, Europe	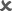
*Echinochloa crus‐galli*	Selfing	No	Medium	Medium	Very high	Hexaploid	Medium, sequenced	North and South America, Australia, Europe, Africa, Asia	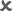

The potential for gene drive systems (green, good potential; yellow, medium; red, low) to provide options for the direct genetic control of weed populations is considered with respect to key weed biological and genetic characteristics, as well as global distribution and difficulty of control (summarized here as herbicide‐resistance risk). The mating system of the weed species determines the inherent capacity for gene drives, because drives will successfully spread only edited genes through outcrossing species. Species are classified as predominantly outcrossing or predominantly selfing. Highly fecund species will most likely have the largest populations, which will extend the time taken for gene drives to result in significant population suppression. Similarly, a high degree of seed bank persistence will slow the rate at which genes proliferate through populations. A very high resistance risk is associated with species that have evolved resistance to more than five modes of action and an intermediate risk to species that have evolved resistance to two or fewer modes of action. For polyploid species and for species with large, unsequenced genomes the identification and targeting of gene targets will be more complex. For the purposes of this analysis, species with genome sizes < 500 Mb were considered to have small genomes, genomes ranging in size from 500 Mb to 2Gb were considered as medium‐sized and those > 2Gb as large genomes

## 6 CONCLUSIONS, PERSPECTIVES AND NEXT STEPS

The considerable excitement that has been generated by recent and rapid developments in gene drive technology merits the attention of those concerned with agricultural weed (and pest) management.^4^, ^5^, ^6^ The need for technological and agroecological innovation in crop protection systems is great, and gene drive offers unprecedented power to directly manipulate the genomes of pest species to: (i) introduce and spread fitness‐reducing traits into wild pest populations to achieve population suppression, and/or (ii) sensitize (or re‐sensitize) populations to new (and existing) control techniques.

The ethical, societal, biosecurity and ecological challenges associated with the use and regulation of gene drives are considerable. Suppression drives represent a form of gene editing that can spread, potentially uncontrollably, through wild populations with the potential to result in severe population declines (and maybe even extinction) of those species in natural systems. There is a rapidly expanding literature that considers these risks and the attendant need for stringent regulation of gene drives^6^, ^45^, ^46^, ^47^ together with a number of methods that have been proposed that safeguard gene drives by limiting their potential spread and/or making them reversible in laboratory and natural environments.^4^, ^31^, ^48^, ^49^ This article is limited to a consideration of the technical feasibility and practical applications of gene drives for weed management, although it should be recognized that the regulatory and ethical barriers to the use of gene drives are likely to be more decisive than any technical difficulties.

The definition of an agricultural pest is anthropogenic, based on the propensity for certain organisms to reduce crop yields via herbivory, disease transmission, pathogenicity and resource competition. In wider context, most pests, including weeds, are integral components of agroecosystems and their undesirability is a function of their abundance, and the associated crop yield loss potential, rather than their inherent presence in those ecosystems. Indeed, many pest species may even perform positive roles in agroecosystems relating to community structure and ecosystem functioning. It is my own personal and scientific view that any gene drive that has the stated goal (or the inherent potential) of driving a species to extinction would not be desirable, and stringent regulations would likely prohibit those aims and potential outcomes. Nevertheless, local eradications may be possible, and it should be noted that gene drives have the potential to enable more targeted pest control that considerably reduces the unintended, off‐target impacts of current crop protection strategies.

Sensitizing drives, particularly those that, for example, reverse the evolution of resistance, may overcome many of the inherent ecological concerns and ethical objections to gene drives. Gene drives to reverse herbicide resistance have inbuilt ‘safeguarding’ mechanisms. At the genetic level, they aim to revert individuals and populations that have evolved resistance via the human‐directed selection of resistance‐conferring alleles to their ancestral wild‐type, sensitive state. As such, these methods do not require the introduction of edited DNA that reduces pest fitness in ‘natural environments’. It could be argued that these drives would restore populations to the genetic state that they were in before human intervention. At the practical level, the population suppression that can be delivered by sensitizing gene drives is by definition coupled tightly to the exogenous application of a chemical (or other management technique) making it possible to strictly limit the spatial and temporal control of populations. It may be the case that these sensitizing drives have the greatest potential to deliver novel, genetic weed control within envisaged future regulatory frameworks.

Technical feasibility and regulatory challenges may ultimately dictate the potential for gene drives to contribute to the future management of agricultural weeds. However, these considerations are only relevant if it can be established that gene drives have the potential to spread effectively through populations in reasonable time frames to affect practical applications. Here, the potential limitations relate to aspects of weed biology, ecology, life history and population dynamics, as well as the potential for populations to rapidly evolve resistance to gene drive systems and/or to the control measures that are enabled by gene editing (see Table 1 and Section 5.1). In many respects, these limitations are the most easily addressed because simulation modelling studies can be used to assess the likely rate and efficacy of spread of gene drives through weed populations under a number of assumptions about the efficiency of homing reactions, release rates, population sizes, weed population dynamics and weed mating systems. As a first step, towards a more rigorous assessment of gene drive potential for weed management, these modelling studies should be undertaken as a priority to inform application domains and to assess the critical factors that will determine success or failure. These modelling studies should also consider how direct genetic control of weeds may be integrated with other chemical, cultural and physical methods of weed management to design gene drive systems that deliver weed control in practicable timescales whilst reducing the potential for rapid evolution of resistance mechanisms. There may be many roadblocks and steering this technology towards application may not ultimately be possible, but given the urgent need for innovation in weed management, it is imperative that all potential future technologies are fully investigated.

## APPENDIX 1

**Table A1:** Definitions

Indirect genetic control	Any genetic control system for an agricultural pest, in which control is delivered via genetic modification (via breeding or transgenesis) of the host or competing crop species genome.
Direct genetic control	Any genetic control system that is based on direct modification of the pest genome to introduce and/or proliferate a fitness‐reducing genetic load.
Gene or meiotic drive	Naturally occurring genetic mechanisms that can propagate a modified gene or suite of genes through an organism's genome by subverting the normal rules of Mendelian inheritance.
Homing endonuclease genes	Selfish genetic elements that are able to propagate through genomes by cleaving chromosomes that do not contain them and are then copied into the broken chromosome during the DNA repair process.
CRISPR–Cas9	A naturally occurring genetic component of the bacterial immune system, now developed as a molecular tool for use in a range of organisms to enable precise genome editing.
Homologous recombination pathway	A mechanism of genetic recombination, whereby nucleotide sequences are replaced by similar or identical (homologous) molecules of DNA to repair DNA double‐strand breaks. The proliferation of CRISPR–Cas9 gene drives through genomes is dependent on this DNA repair pathway.
Non‐homologous end joining pathway	An alternative pathway for the repair of this DNA double‐strand breaks in which the broken ends are directly ligated without the need for a homologous DNA template.
